# Endoscopic and Histopathologic Findings of Rituximab-Associated Colitis in a Patient With Scleroderma

**DOI:** 10.7759/cureus.88780

**Published:** 2025-07-25

**Authors:** Roberto A Alvarado-Hernández, Juan M Reyes-Morales, Yoeli M Escandon-Espinoza, Katia D Lopez Garcia, Victor M Ayuso-Diaz, Angelica Moreno-Enriquez

**Affiliations:** 1 Gastrointestinal Endoscopy, Institute for Social Security and Services for State Workers (ISSSTE) - Hospital Regional De Alta Especialidad Bicentenario De La Independencia, Tultitlan, MEX; 2 Research and Education Division, Medical Care and Research, Mérida, MEX; 3 Genomic-Metabolic Unit, Marista University of Merida, Mérida, MEX

**Keywords:** b-cell regulation, biologic-induced colitis, endoscopic findings, histopathologic colitis, inflammatory bowel disease, interleukin 10, rituximab toxicity, scleroderma, therapeutic endoscopy, ulcerative colitis

## Abstract

Rituximab is a chimeric monoclonal antibody that targets the CD20 antigen found on B lymphocytes. It is widely used in the treatment of haematological malignancies and, more recently, autoimmune diseases such as rheumatoid arthritis and scleroderma. While its safety profile is generally considered to be acceptable, a range of gastrointestinal adverse effects have been reported, including colitis. While this event is uncommon, there is a documented association with the development of de novo inflammatory bowel disease, particularly in patients with no prior history of digestive pathology. The clinical manifestations may be subtle or overlap with symptoms commonly found in other autoimmune diseases, which makes diagnosis difficult and increases the risk of complications such as stenosis, haemorrhage, or perforation.

We present the case of a patient with systemic sclerosis under chronic rituximab treatment who developed colitis with clinical, endoscopic, and histopathological features consistent with ulcerative colitis. This case highlights the need for clinicians to remain vigilant for persistent gastrointestinal symptoms in patients undergoing anti-CD20 therapy and underscores the importance of early endoscopic evaluation with biopsies for the prompt detection of such complications.

## Introduction

Rituximab is a chimeric IgG1 monoclonal antibody that binds to the CD20 antigen found on pre-B and mature B lymphocytes. Its mechanisms of action include complement-dependent cytotoxicity, antibody-dependent cellular cytotoxicity, and apoptosis [1,2]. Although rituximab was initially developed to treat B-cell lymphomas, its potent immunomodulatory effects have expanded its use to various autoimmune diseases such as rheumatoid arthritis, systemic lupus erythematosus, and systemic sclerosis (scleroderma) [3].

However, sustained B-cell depletion induced by rituximab may have unforeseen immunological consequences [2]. An emerging body of evidence suggests an association between prolonged rituximab use and the development of de novo inflammatory bowel disease (IBD), including ulcerative colitis and Crohn’s disease, particularly in patients without prior gastrointestinal pathology [4,5]. In a retrospective cohort study conducted at the Mayo Clinic, histologically confirmed cases of inflammatory colitis were identified in up to 5% of patients who developed diarrhoea following rituximab administration, despite no prior history of IBD [6].

From a mechanistic perspective, rituximab-induced disruption of B-cell-mediated immune regulation is thought to impair intestinal homeostasis. Regulatory B cells (Bregs), particularly those producing interleukin-10 (IL-10), play a central role in mucosal tolerance by suppressing proinflammatory responses and supporting epithelial integrity. Their sustained depletion may predispose to dysbiosis, epithelial injury, and chronic intestinal inflammation, thus triggering IBD in genetically or immunologically susceptible individuals.

## Case presentation

A 42-year-old female patient with an eight-year history of limited systemic sclerosis, predominantly affecting the skin of her hands, forearms, face, and oesophagus, was referred for gastrointestinal evaluation due to persistent digestive symptoms. She had been receiving intravenous rituximab (1 g every six months) as chronic immunosuppressive therapy for the past five years, resulting in favourable systemic control and stabilisation of autoimmune parameters.

Her medical history included a documented hiatal hernia and oesophageal stricture. She had previously undergone five endoscopic dilatation sessions using a controlled radial expansion balloon due to persistent dysphagia. Biopsies taken during these procedures revealed non-specific chronic oesophagitis, without evidence of eosinophilic infiltration, intestinal metaplasia, or features suggestive of IBD.

Approximately three years prior to the current consultation, the patient began experiencing intermittent episodes of diarrhoea and constipation, occasionally accompanied by rectal bleeding. These symptoms were not associated with fever, weight loss, or other systemic signs of inflammation. On one occasion, she developed colicky abdominal pain, distension, and absence of bowel movements for 48 hours, which resolved with conservative management and did not require hospitalisation. Although initially attributed to functional bowel symptoms, the recurrence of similar gastrointestinal complaints in the current evaluation prompted further investigation.

**Figure 1:**
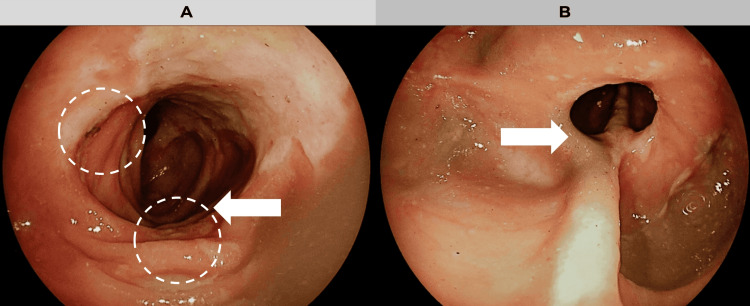
Colonoscopy. A. Endoscopic view of the descending colon showing two areas of mucosal ulceration (highlighted with dotted circles), with irregular borders and adjacent oedematous mucosa. B. Severe stenosis at the transverse colon, with significant luminal narrowing and mucosal irregularity.

Due to the chronicity of her symptoms and a positive faecal occult blood test, an elective colonoscopy was performed to rule out neoplasia or IBD. Faecal calprotectin testing was not available at the time. During colonoscopy, multiple linear and serpiginous ulcers were observed in the rectum, sigmoid, and descending colon, alongside two significant strictures in the transverse colon, affecting 30% and 60% of the lumen, respectively (Figures 1A, 1B). Targeted biopsies were taken from both the ulcerated and stenotic areas for histopathological analysis. Despite technical challenges in photographing the lesions due to colonic angulation and limited distension, endoscopic inspection confirmed the presence of extensive, irregular ulcers with oedematous borders and surrounding mucosal inflammation (Figure 1A).

During the clinical evaluation, the patient was afebrile and haemodynamically stable, with no signs of significant weight loss or overt gastrointestinal bleeding. Abdominal examination revealed a soft, non-tender abdomen with normal bowel sounds present. No organomegaly or masses were detected.

**Figure 2:**
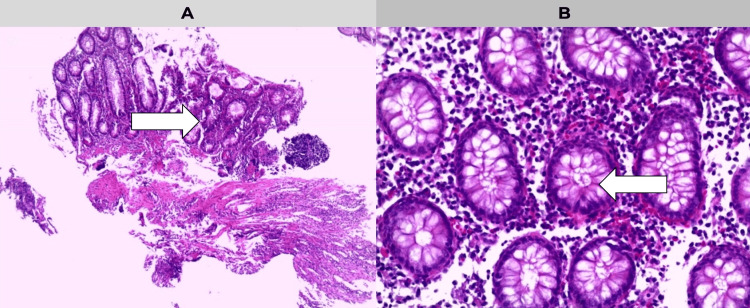
Histopathological findings compatible with chronic active colitis. A. Low-magnification image (H&E, 10×) showing architectural distortion of the colonic crypts, decreased glandular density, and evident subepithelial fibrosis, as well as mucosal disorganisation — all typical findings of chronic colitis. Areas of transmucosal inflammation without granuloma formation can also be seen. B. High-magnification image (H&E, 40×) showing elongated and branching crypts with Paneth cell metaplasia and a chronic inflammatory infiltrate composed mainly of lymphocytes and plasma cells in the lamina propria, as well as evidence of active cryptitis.

Following colonoscopy, histopathological analysis revealed features consistent with active chronic colitis. These included architectural distortion of the crypts, a dense lymphoplasmacytic infiltrate, Paneth cell metaplasia, subepithelial fibrosis, and basal plasmacytosis. There was no evidence of granulomas, vasculitis, or foreign bodies, findings which effectively ruled out Crohn’s disease and other differential diagnoses. Taken together, the absence of transmural inflammation and granulomas, alongside the distribution and microscopic features, supported a definitive diagnosis of ulcerative colitis, a subtype of IBD (Figures 2A, 2B).

## Discussion

This case highlights an emerging and clinically relevant complication of long-term rituximab therapy in patients with systemic autoimmune diseases: the development of de novo inflammatory colitis with endoscopic and histopathological features closely resembling those of IBD. Identifying extensive ulcerations, inflammatory colonic strictures, and mucosal biopsy findings consistent with chronic active colitis in a patient with no prior gastrointestinal symptoms highlights the importance of increased clinical vigilance in individuals undergoing B-cell-depleting therapies [4].

While gastrointestinal involvement, including dysmotility and mucosal abnormalities, is well recognised in systemic sclerosis, the simultaneous presence of multiple serpiginous ulcers and inflammatory strictures, particularly in the transverse colon, is atypical. Furthermore, the histological architecture observed in this case lacked features characteristic of scleroderma enteropathy, such as fibrosis and atrophy without active inflammation, and instead showed hallmark signs of ulcerative colitis, such as crypt distortion, Paneth cell metaplasia, and a lymphoplasmacytic infiltrate [5,6]. These findings suggest a distinct pathophysiological mechanism, likely driven by the immunomodulatory effects of rituximab.

From a mechanistic perspective, Bregs, including those that produce interleukin-10 (IL-10), play a key role in maintaining intestinal immune tolerance. Bregs promote epithelial integrity, support the production of secretory IgA, and suppress the activation of pro-inflammatory Th1 and Th17 cells [4,7]. However, sustained depletion of CD20+ B cells by rituximab disrupts this immunological equilibrium, resulting in dysbiosis, epithelial barrier dysfunction, and enhanced mucosal inflammation. While this shift is beneficial for controlling systemic autoimmunity, it paradoxically increases the risk of chronic intestinal inflammation, a mechanism also implicated in IBD pathogenesis [7,8].

Rituximab has been investigated as a treatment for refractory ulcerative colitis, but clinical trials and case series have shown inconsistent results, with some patients experiencing a paradoxical worsening of their condition [6,9]. This paradox may be explained by the persistence of CD20-negative mucosal plasma cells, which continue to fuel immune dysregulation despite peripheral B-cell depletion [9].

In this context, endoscopy is useful not only for anatomical assessment but also for enabling histopathological sampling, which is critical for distinguishing between primary IBD, drug-induced injury, and autoimmune enteropathy [10]. While not a novel diagnostic modality, endoscopy remains essential in evaluating atypical or persistent symptoms in patients on immunomodulators.

Although gastrointestinal toxicity from rituximab is uncommon, clinicians should be aware of this potential adverse effect and promptly initiate a gastrointestinal evaluation in symptomatic patients. While routine screening of all patients receiving rituximab may not be justified, awareness of this condition may prompt timely referral and intervention when symptoms such as rectal bleeding or chronic diarrhoea develop [11].

Simple, symptom-based algorithms incorporating faecal occult blood testing or non-invasive inflammatory markers may help to stratify risk and identify patients requiring endoscopic evaluation [12].

This report is limited by the absence of colonoscopic data prior to rituximab initiation, which precludes definitive causal attribution. While the clinical, endoscopic, and histological features strongly suggest rituximab-associated colitis, alternative explanations such as subclinical IBD, scleroderma colopathy, or coincidental IBD onset cannot be completely ruled out. Prospective multicentre studies are urgently needed to determine the incidence, clinical predictors, and immunological characteristics of this condition, and to explore preventive strategies, such as preserving regulatory B-cell subsets or restoring intestinal tolerance through adjunctive therapies. Nevertheless, this report adds valuable evidence to the growing body of literature on rituximab-associated enteropathies.

## Conclusions

Rituximab-induced colitis remains a rare but clinically relevant complication in patients with systemic autoimmune diseases. This case underscores the importance of maintaining a high index of suspicion in patients presenting with persistent gastrointestinal symptoms during anti-CD20 therapy. Early endoscopic evaluation with targeted biopsies is essential to establish a timely and accurate diagnosis, thereby preventing serious complications such as strictures or haemorrhage.

Although often overlooked, rituximab-associated colitis may reflect an immune-mediated process involving disruption of intestinal homeostasis. This highlights the potential role of regulatory B cells in maintaining mucosal integrity and the need to consider gastrointestinal risks when selecting immunosuppressive regimens. Personalised medicine should weigh the systemic benefits of rituximab against its impact on mucosal immunity. In the present case, the patient responded favourably to appropriate treatment following diagnosis, with gradual resolution of symptoms and no further gastrointestinal complications during follow-up.
